# Phase separation of hnRNPA1 and TERRA regulates telomeric stability

**DOI:** 10.1093/jmcb/mjae037

**Published:** 2024-09-23

**Authors:** Ziyan Xu, Yongrui Liu, Fudong Li, Yi Yang, Hong Zhang, Feilong Meng, Xing Liu, Xin Xie, Xianjun Chen, Yunyu Shi, Liang Zhang

**Affiliations:** Center for Advanced Interdisciplinary Science and Biomedicine of IHM, Division of Life Sciences and Medicine, University of Science and Technology of China, Hefei 230027, China; Ministry of Education Key Laboratory for Membraneless Organelles and Cellular Dynamics, Division of Life Sciences and Medicine, University of Science and Technology of China, Hefei 230027, China; Hefei National Research Center for Cross-disciplinary Science, Division of Life Sciences and Medicine, University of Science and Technology of China, Hefei 230027, China; Center for Advanced Interdisciplinary Science and Biomedicine of IHM, Division of Life Sciences and Medicine, University of Science and Technology of China, Hefei 230027, China; Ministry of Education Key Laboratory for Membraneless Organelles and Cellular Dynamics, Division of Life Sciences and Medicine, University of Science and Technology of China, Hefei 230027, China; Hefei National Research Center for Cross-disciplinary Science, Division of Life Sciences and Medicine, University of Science and Technology of China, Hefei 230027, China; Center for Advanced Interdisciplinary Science and Biomedicine of IHM, Division of Life Sciences and Medicine, University of Science and Technology of China, Hefei 230027, China; Ministry of Education Key Laboratory for Membraneless Organelles and Cellular Dynamics, Division of Life Sciences and Medicine, University of Science and Technology of China, Hefei 230027, China; Hefei National Research Center for Cross-disciplinary Science, Division of Life Sciences and Medicine, University of Science and Technology of China, Hefei 230027, China; Optogenetics and Synthetic Biology Interdisciplinary Research Center, State Key Laboratory of Bioreactor Engineering, East China University of Science and Technology, Shanghai 200237, China; National Laboratory of Biomacromolecules, CAS Center for Excellence in Biomacromolecules, Institute of Biophysics, Chinese Academy of Sciences, Beijing 100101, China; College of Life Sciences, University of Chinese Academy of Sciences, Beijing 100049, China; State Key Laboratory of Molecular Biology, Shanghai Institute of Biochemistry and Cell Biology, Center for Excellence in Molecular Cell Science, Chinese Academy of Sciences, University of Chinese Academy of Sciences, Shanghai 200031, China; Center for Advanced Interdisciplinary Science and Biomedicine of IHM, Division of Life Sciences and Medicine, University of Science and Technology of China, Hefei 230027, China; Ministry of Education Key Laboratory for Membraneless Organelles and Cellular Dynamics, Division of Life Sciences and Medicine, University of Science and Technology of China, Hefei 230027, China; Hefei National Research Center for Cross-disciplinary Science, Division of Life Sciences and Medicine, University of Science and Technology of China, Hefei 230027, China; Optogenetics and Synthetic Biology Interdisciplinary Research Center, State Key Laboratory of Bioreactor Engineering, East China University of Science and Technology, Shanghai 200237, China; Optogenetics and Synthetic Biology Interdisciplinary Research Center, State Key Laboratory of Bioreactor Engineering, East China University of Science and Technology, Shanghai 200237, China; Center for Advanced Interdisciplinary Science and Biomedicine of IHM, Division of Life Sciences and Medicine, University of Science and Technology of China, Hefei 230027, China; Ministry of Education Key Laboratory for Membraneless Organelles and Cellular Dynamics, Division of Life Sciences and Medicine, University of Science and Technology of China, Hefei 230027, China; Hefei National Research Center for Cross-disciplinary Science, Division of Life Sciences and Medicine, University of Science and Technology of China, Hefei 230027, China; Center for Advanced Interdisciplinary Science and Biomedicine of IHM, Division of Life Sciences and Medicine, University of Science and Technology of China, Hefei 230027, China; Ministry of Education Key Laboratory for Membraneless Organelles and Cellular Dynamics, Division of Life Sciences and Medicine, University of Science and Technology of China, Hefei 230027, China; Hefei National Research Center for Cross-disciplinary Science, Division of Life Sciences and Medicine, University of Science and Technology of China, Hefei 230027, China

**Keywords:** phase separation, crystal structure, telomeric stability, protein–RNA complex, hnRNPA1, TERRA

## Abstract

Telomeres are the complexes composed of repetitive DNA sequences and associated proteins located at the end of chromatin. As a result of the DNA replication ending issue, telomeric DNA shortens during each cell cycle. The shelterin protein complex caps telomeric ends and forms a high-order protein–DNA structure to protect telomeric DNA. The stability of telomeres is critical for cellular function and related to the progression of many human diseases. Telomeric repeat-containing RNA (TERRA) is a noncoding RNA transcribed from telomeric DNA regions. TERRA plays an essential role in regulating and maintaining the stability of telomeres. Heterogeneous nuclear ribonucleoproteins (hnRNPs) are RNA-binding proteins associated with complex and diverse biological processes. hnRNPA1 can recognize both TERRA and telomeric DNA. Previous research reported that hnRNPA1, TERRA, and POT1, a component of the shelterin complex, work coordinately and displace replication protein A from telomeric single-stranded DNA after DNA replication, promoting telomere capping to preserve genomic integrity. However, the detailed molecular mechanism has remained unclear for >20 years. Here, our study revealed the molecular structure through which the hnRNPA1 UP1 domain interacts with TERRA and identified critical residues on the interacting surface between UP1 and TERRA. Furthermore, we proved that nucleic acids significantly increase the phase-separating ability of hnRNPA1, while disrupting the UP1–TERRA interaction extraordinarily affects hnRNPA1 droplet formation both *in vitro* and *in vivo*. Taken together, these data reveal the molecular mechanism of the phase separation of hnRNPA1 and TERRA and the potential contribution of the droplets to maintaining genomic stability.

## Introduction

The mammalian cell cycle is divided into mitosis (M) phase, DNA synthesis (S) phase, and gap phases G_1_ and G_2_. After the duplication of chromatin in S phase, repetitive DNA sequences and associated proteins form protective caps at the end of each chromosome to assemble the telomeres (Maciejowski and de Lange, 2017; de Lange, 2018). Together with centromeres, machinery connecting chromosomes to the mitotic spindle (Cleveland et al., 2003; Liu et al., 2020), telomeres maintain genomic integrity, prevent the loss of genetic information during replication, and play vital roles in cell division and overall cellular health (Kratz and de Lange, 2018; Borges et al., 2022; Denham, 2023). During each round of DNA replication, a small portion of the telomeric DNA is unavoidably lost due to the end replication problem (Rossiello et al., 2022; Veschetti et al., 2023). The cell will die when the telomere is shorter than a critical length. Therefore, maintaining and guiding orderly telomere replication and proper capping are essential for telomeric stability. The protection of telomeres 1 (POT1) protein is a critical component of the shelterin complex and helps to protect the telomeric overhang and prevent unwanted DNA repair activities at the telomere (Wang et al., 2007; Chen et al., 2017). Replication protein A (RPA), a eukaryotic single-stranded DNA (ssDNA)-binding protein, is essential for both DNA replication and repair (Wold, 1997; Sui et al., 2022). The timely replacement of RPA by POT1 on the telomeric overhang to upgrade shelterin assembly is extremely important for maintaining genomic stability. Previous research indicated that telomeric repeat-containing RNA (TERRA) and heterogeneous nuclear ribonucleoprotein A1 (hnRNPA1) orchestrated the POT1–RPA switch, but the molecular detail remains unclear (Flynn et al., 2011).

**Figure 1:**
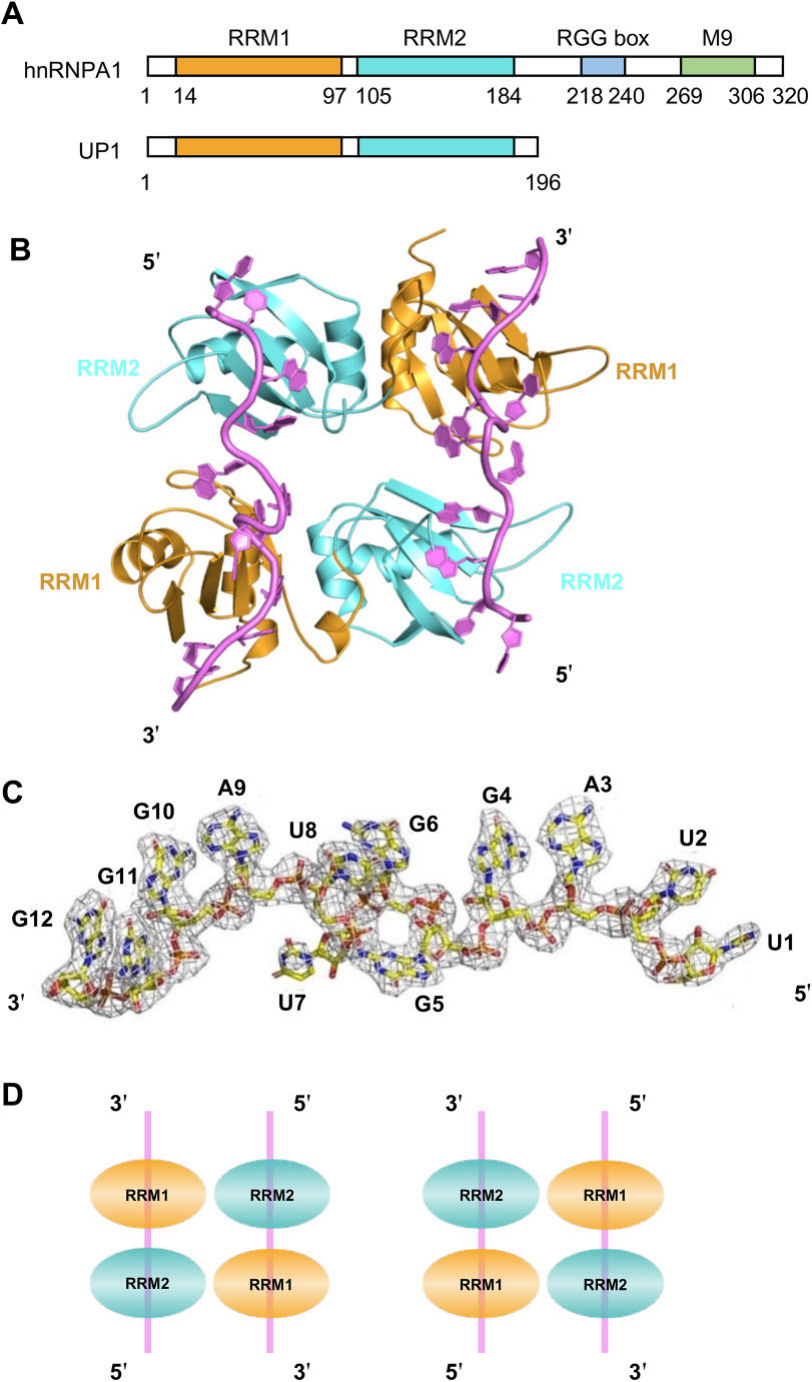
Structure of the UP1–TERRA complex. (**A**) Schematic representation of the sequence distribution of hnRNPA1 and UP1. (**B**) Ribbon overview of the crystal structure of UP1–TERRA. The TERRA fragments are colored pink, and the RRM1 and RRM2 domains of UP1 are colored yellow and cyan, respectively. (**C**) Electron density map of TERRA fragments. The RNA is represented as a yellow ribbon. The 2*F*o–*F*c omit density map of the TERRA fragment was contoured at 1.0 σ. (**D**) The model shows the behaviors of UP1 interacting with TERRA repeats (left) and telomeric DNA repeats (right).

TERRA is a noncoding RNA molecule transcribed from several telomeric DNA regions (Luke and Lingner, 2009; Kroupa et al., 2022; Perez-Martinez et al., 2022). It has been reported that TERRA plays essential roles in telomere maintenance and regulation through different mechanisms, e.g. inhibition of TERRA transcription decreases both DNA replication stress and DNA damage marks at telomeres and impairs telomere length maintenance (Lalonde and Chartrand, 2020; Silva et al., 2021). In addition, hnRNPs are RNA-binding proteins associated with a lot of complex and diverse biological processes, such as the processing of hnRNAs into mature mRNAs, RNA splicing, and modulation of protein translation (Martinez-Contreras et al., 2007; Geuens et al., 2016; Liu and Shi, 2021). hnRNPA1 is the most abundant and ubiquitously expressed member of this protein family and involved in multiple molecular events driving malignant transformation (Roy et al., 2017). hnRNPA1 comprises two tandem RRM domains (UP1), which take charge of nucleic acid recognition, and a C-terminal low complexity domain containing an RGG box, which has been implicated in the formation of stress granules through liquid–liquid phase separation (LLPS) (Figure 1A; Kattimani and Veerappa, 2018; Zhao et al., 2018; Baradaran-Heravi et al., 2020). hnRNPA1 was initially found to be a DNA-binding protein implicated in telomere maintenance. Notably, telomeres consist of tandemly repeated short DNA sequences that can be different in various organisms, and the hexanucleotide sequence is TTAGGG in mammals. Previous research reported the crystal structure of UP1 in complex with telomeric ssDNA (Ding et al., 1999). The sequence of mammalian TERRA is the UUAGGG repeats, while TERRA is transcribed from the telomere region. Recent studies have indicated that the shelterin complex regulates telomere replication and chromosome end-capping, while disease-causing mutations in their encoding genes may affect specific functions (Lim and Cech, 2021). hnRNPA1, TERRA, and POT1 act in concert to displace RPA from telomeric ssDNA following DNA replication and promote telomere capping to preserve genomic integrity (Flynn et al., 2011). RPA replacing is inhibited by TERRA in the early S phase but unleashed in the late S phase when TERRA levels decline at telomeres (Porro et al., 2010; Redon et al., 2013; Hirashima and Seimiya, 2015).

Interestingly, TERRA also promotes POT1 binding to telomeric ssDNA by removing hnRNPA1, suggesting that the re-accumulation of TERRA after S phase helps to complete the RPA-to-POT1 switch on telomeric ssDNA (Sui et al., 2015). However, the exact mechanism by which TERRA binds with hnRNPA1 to displace it from the telomeres remains unclear. Additionally, it is still uncertain how hnRNPA1 is prevented from re-binding with telomeric DNA in competition with POT1. These molecular processes require further investigation.

In our study, by solving the crystal structure of the hnRNPA1 UP1–TERRA complex and investigating hnRNPA1 droplet formation, we revealed the molecular mechanism by which hnRNPA1 recognizes TERRA and regulates the genomic stability and telomere capping both *in vitro* and *in vivo*.

## Results

### The mode of the UP1 domain binding with TERRA

hnRNPA1 comprises an N-terminal UP1 domain (residues 14–184, tandemly linked RRM1 and RRM2) and a C-terminal intrinsic disorder domain that contains an RGG box and a nuclear localization sequence (residues 196–372) (Figure 1A). Previous research reported that hnRNPA1 interacts with TERRA *in vivo* through multiple modes (Le et al., 2013). Further investigation revealed that the UP1 domain is sufficient for binding with two repeats of TTAGGG sequence (Ding et al., 1999) and the RGG box could recognize the TERRA G-quadruplexes *in vitro* (Ghosh and Singh, 2020). To understand how UP1 interacts with telomeric RNA, the crystal structure of the UP1–TERRA complex was solved at a resolution of 2.80 Å (Table 1). Overall, two strands of (UUAGGG)_2_ RNA bind with two UP1 molecules in the complex structure (Figure 1B). The two RNA molecules are antiparallel, i.e. each 5′ terminus is located close to RRM2 of one UP1 monomer, while the 3′ terminus is located near RRM1 of the other UP1 monomer. The electron density map of RNA molecules describes the contents of the unit cells averaged over the whole crystal rather than the contents of a single unit cell (Figure 1C). Moreover, in the UP1–TERRA complex and the UP1–telomeric ssDNA repeat complex (PDB code: 2UP1), the binding orientations of the polypeptide chain and the nucleic acid chain were opposite (Figure 1D). RRM1 of UP1 always binds with the 3′ end of TERRA or the 5′ end of telomeric DNA, while RRM2 of UP1 binds with the 5′ terminus of TERRA or the 3′ terminus of telomeric DNA.

**Table 1:** Data collection and refinement statistics for the UP1–TERRA complex.

	UP1–TERRA
**Data Collection**	
Wavelength (Å)	0.9789
Space group	*P*4_3_2_1_2
PDB code	8X0N
Cell parameters	
a, b, c (Å)	101.30, 101.30, 55.34
α, β, γ (°)	90.00, 90.00, 90.00
Resolution* (Å)	28.82–2.80 (2.85–2.80)
Rmerge (%)	15.8 (52.3)
*I*/σ*I*	9 (3)
Completeness (%)	99.9 (99.7)
Redundancy	5.3 (5.7)
**Refinement**	
No. reflections used/free	7075/548
Resolution (Å)	28.82–2.80
*R*_work_/*R*_free_(%)	23.44/28.97
R.m.s.deviations	
Bond lengths (Å)	0.002
Bond angles (°)	0.458
*B*-factors (Å^2^)	
Protein	45.07
RNA	55.49
Water	NA
No. atoms	
Protein	1449
RNA	259
Water	NA
Ramachandran plot	
Favored/allowed/outlier (%)	97.77/2.23/0

*Values in parentheses are for the highest-resolution shell.

### The critical residues involved in UP1–TERRA interactions

**Figure 2:**
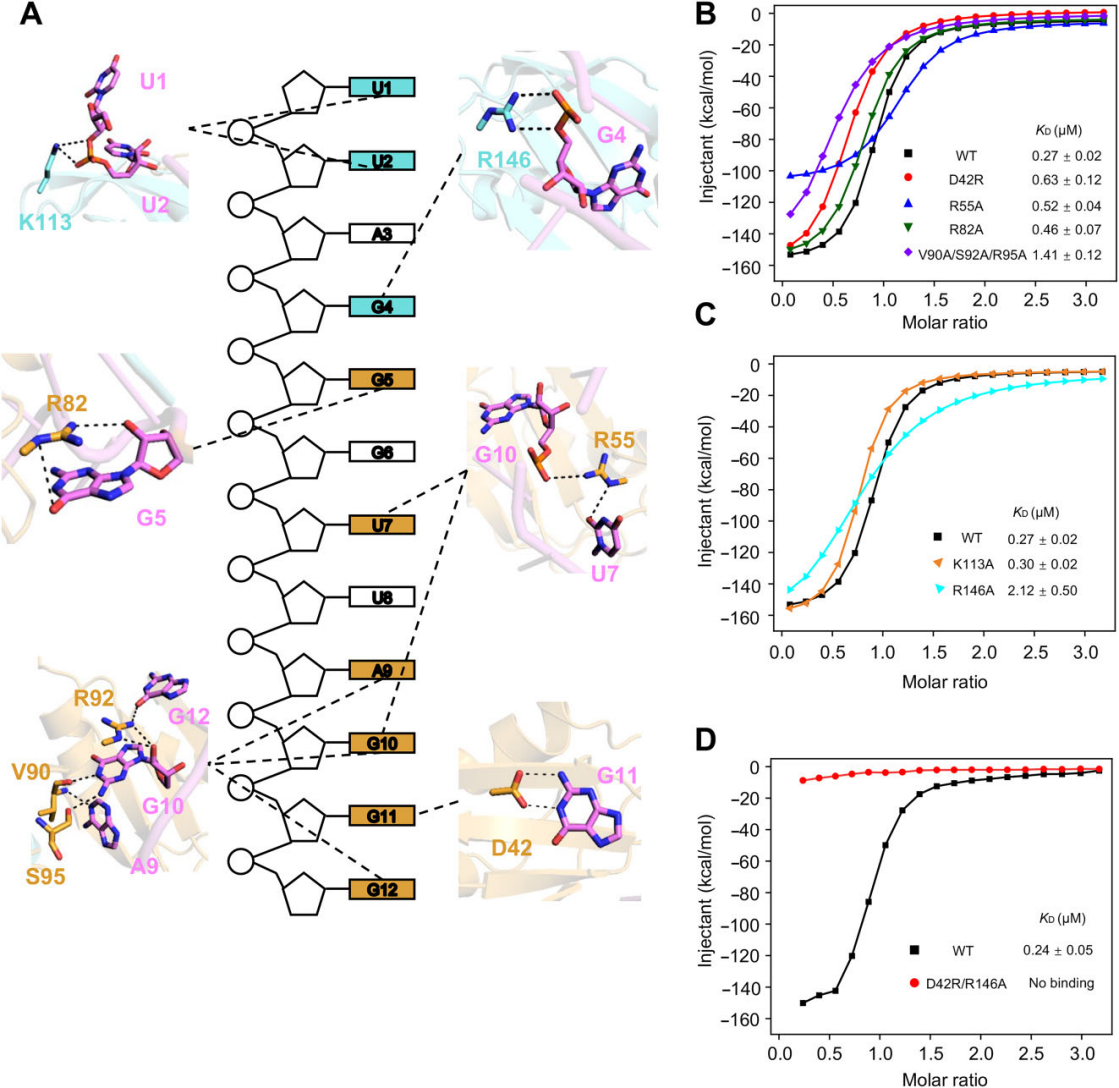
Structural basis of UP1 recognition of the TERRA fragment. (**A**) The interaction details of TERRA with surrounding residues of UP1 in the UP1–TERRA complex. (**B**) ITC fitting curves of wild-type (black squares) and mutant (D42R as red circles, R55A as blue triangles, R82A as green triangles, and V90A/S92A/R95A triple mutation as purple diamonds) RRM1 from UP1 to the 12-bp TERRA repeats. (**C**) ITC fitting curves of wild-type (black squares) and mutant (K113A as orange triangles and R146A as cyan triangles) RRM2 from UP1 to the 12-bp TERRA repeats. (**D**) ITC fitting curves of wild-type (black squares) and mutant (D42R/R146A as red cycles) hnRNPA1 to the 12-bp TERRA repeats.

The interface of RRM1 and the 3′ terminal RNA is contributed by multiple interactions: six amino acids from RRM1 of UP1, Asp42, Arg55, Arg82, Val90, Arg92, and Ser95, contact six different bases of the 3′ end of TERRA (Figure 2A). Asp42 donates two hydrogen bonds with the guanine group of G11, forming a bidentate interaction. A9, G10, and G12 of RNA combine with Arg55, Val90, Arg92, and Ser95 from RRM1, creating a hydrogen network to strengthen UP1–TERRA interactions. In detail, Val90 forms hydrogen-bonding interactions with the basic groups of A9 and G10. Furthermore, G10 interacts with Arg92 through its sugar ring and Ser95 through its guanine group. The side chain of Arg92 also forms a hydrogen bond with the guanine group of G12, and the side chain of Arg55 contacts the phosphate group of G10. In addition, the side chain of Arg55 interacts with U7 through its uracil group, and the side chain of Arg82 forms hydrogen bonds with the sugar ring and guanine group of G5, which is from the first copy of the TERRA repeat on the 5′ terminus.

The interactions of the second RRM (RRM2) of UP1 are relatively less. The side chain of Lys113 donates two hydrogen bonds to the phosphate group of U2, and the side chain of Arg146 forms two hydrogen bonds with the phosphate group of G4.

By aligning and comparing the structures of UP1–TERRA and UP1–telomeric ssDNA, we found that most of the interactions between the UP1 residues and nucleic acids are shared, except that Arg82 from RRM1 and Lys113 from RRM2 only interact with RNA fragment, while Gln12 from RRM1 and Glu147 from RRM2 only have contacts with DNA (Supplementary Figure S1). The

side chain of Gln12 contacts the guanine group of G4, and the Gly147 interacts with the sugar ring of T8 through its carbonyl group.

### Mutations in the UP1–TERRA interface disrupt protein–RNA binding

To confirm the interacting pattern of UP1–TERRA, mutations of critical residues of UP1 and isothermal titration calorimetry (ITC) measurements were carried out. Each point mutation of an amino acid affected UP1–TERRA binding to varying degrees (Table 2). Notably, only mutating the residues within one RRM did not significantly reduce the binding ability to the substrate RNA fragment (Figure 2B and C; Supplementary Figure S2A and B), probably due to the multiple interactions between the protein and RNA. D42A, R146A, and triple mutant (V90A/R92A/S95A) reduced the binding affinity between UP1 and TERRA more severely, with the *K*_D_ values 3, 10, and 7 times that of wild-type UP1, respectively. As expected, when we mutated both RRMs of UP1 (D42R/R146A), the mutant could not bind to RNA at all (Figure 2D; Supplementary Figure S2C).

**Table 2:** ITC results.

Protein	Nucleic acids	*△H* (kcal/mol)	*−T△S* (kcal/mol)	*N*	*K*_D_ (μM)
UP1^wt^	TERRA*	−36.8 ± 0.42	28.0	0.86	0.27 ± 0.02
UP1^D42R^	TERRA	−39.7 ± 1.90	31.3	0.62	0.63 ± 0.12
UP1^R55A^	TERRA	−24.6 ± 0.33	16.3	1.12	0.52 ± 0.04
UP1^R82A^	TERRA	−37.3 ± 1.11	28.9	0.77	0.46 ± 0.07
UP1^V90A/R92A/S95A^	TERRA	−39.9 ± 1.54	32.3	0.51	1.41 ± 0.12
UP1^K113A^	TERRA	−37.8 ± 0.34	29.2	0.72	0.30 ± 0.02
UP1^R146A^	TERRA	−42.5 ± 3.92	34.9	0.83	2.12 ± 0.50
UP1^wt^	TERRA	−36.8 ± 0.42	28.0	0.86	0.27 ± 0.02
UP1^wt^	Telo DNA**	−38.5 ± 0.80	28.9	0.82	0.08 ± 0.02
UP1^D42R/R146A^	TERRA	−30.6 ± 1.68	23.0	0.87	2.26 ± 0.31
UP1^D42R/R146A^	Telo DNA	−44.0 ± 9.83	36.6	0.58	4.03 ± 1.02
UP1^R82A/K113A^	TERRA	−40.9 ± 1.80	32.5	1.18	0.77 ± 0.16
UP1^R82A/K113A^	Telo DNA	−32.1 ± 1.13	22.4	0.94	0.10 ± 0.03
UP1^Q12A/G147A^	TERRA	−41.4 ± 0.65	31.8	0.72	0.12 ± 0.02
UP1^Q12A/G147A^	Telo DNA	−17.9 ± 0.94	8.8	0.77	0.21 ± 0.08

*The sequence of TERRA is 5′-UUAGGGUUAGGG-3′.

**The sequence of Telo DNA is 5′-TTAGGGTTAGGG-3′.

To understand the binding specificities of UP1 to TERRA and telomeric ssDNA, we also generated RNA-specific and DNA-specific mutants. The RNA-related double mutant (R82A/K113A) reduced the binding affinity between UP1 and the TERRA fragment by ∼3 times without affecting the DNA binding affinity, while the DNA-specific double mutant (Q12A/G147A) also lowered the binding affinity between UP1 and DNA by 2.6 times (Table 2).

### RNA promotes the phase separation of hnRNPA1

**Figure 3:**
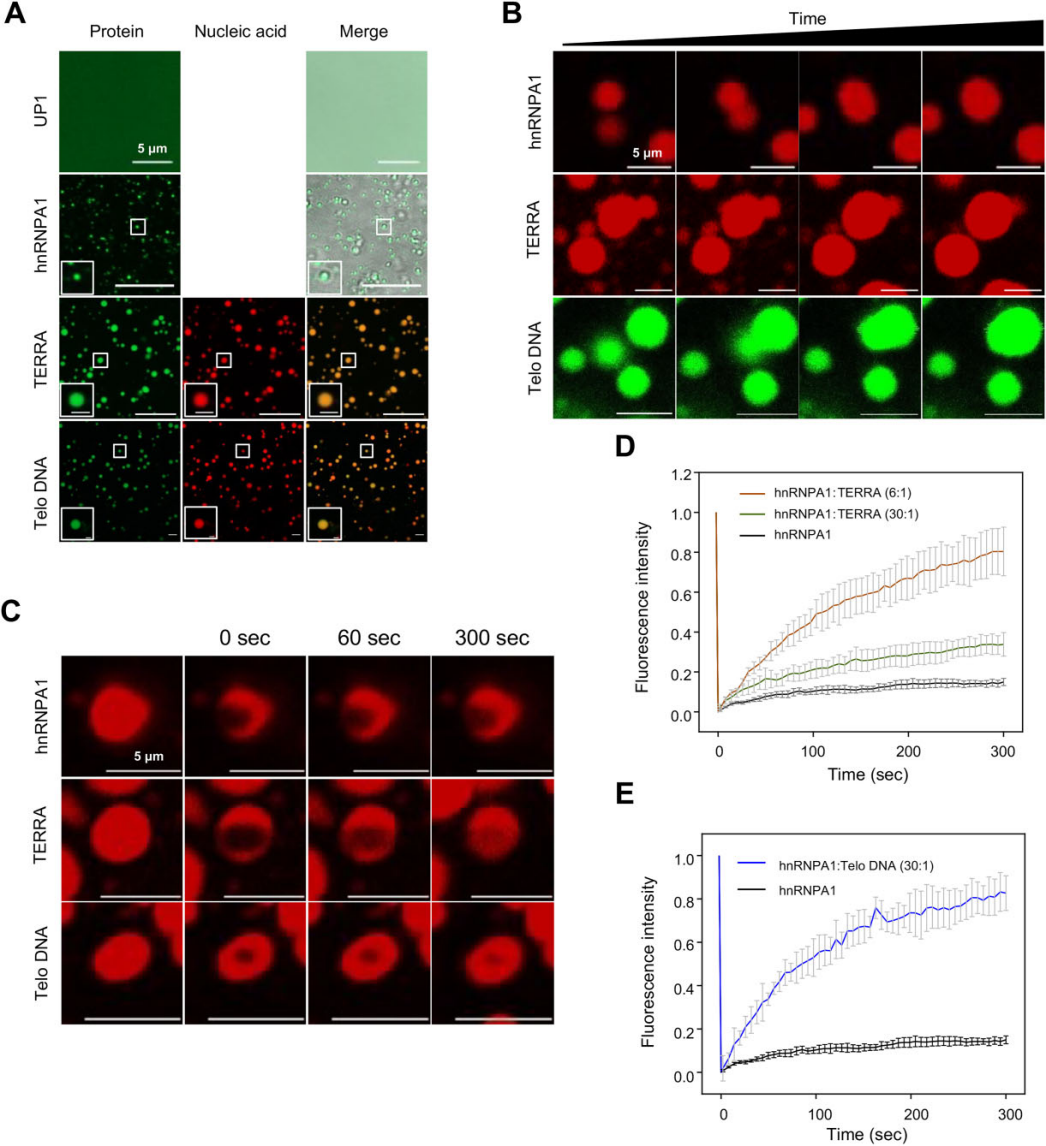
LLPS analysis of wild-type hnRNPA1 *in vitro*. (**A**) Phase separation analysis of hnRNPA1 and UP1 using fluorescence microscopy. Protein was partially (5%) labeled with Alexa Fluor 488, and nucleic acid was labeled with Cy3 dye. The sequence of TERRA was 5′-UUAGGGUUAGGG-3′, and the telomeric ssDNA (Telo DNA) sequence was 5′-TTAGGGTTAGGG-3′. The molar ratio of hnRNPA1 to nucleic acid was 30:1. Scale bar, 5 μm. (**B**) Phase-separated liquid droplets by hnRNPA1 alone or hnRNPA1 with nucleic acids fuse and form a larger droplet. Scale bar, 5 μm. (**C**) Snapshots of a bleached droplet of hnRNPA1 alone or hnRNPA1 with TERRA or Telo DNA at different time points. Scale bar, 5 μm. (**D** and **E**) Quantification of average fluorescence intensities in **C**.

hnRNPA1 contains a C-terminal intrinsically disordered region that drives hnRNPA1 to phase-separate and join into stress granules under stress conditions (Molliex et al., 2015). To determine whether the UP1 region or RNA substrate contributes to the formation and properties of hnRNPA1 droplets, we examined droplet formation of the full-length hnRNPA1 protein complexed with different nucleic acid fragments. As shown in Figure 3A, the UP1 domain alone cannot form the granule, but the full-length hnRNPA1 can, and TERRA and telomeric ssDNA can co-separate with the full-length hnRNPA1 protein. To investigate the liquid-like properties of the granules, we captured the fusing process of the granules formed by hnRNPA1 and different nucleic acids (Figure 3B). The phase-separated puncta formed by hnRNPA1, either with or without nucleic acids, all fused into large droplets in a few seconds. However, the fluorescence recovery after photobleaching (FRAP) experiments demonstrated that the addition of nucleic acids can significantly increase the fluidity of the hnRNPA1 droplets, i.e. higher exchange rates as well as quicker and better fluorescence recovery (Figure 3C–E).

### RRM mutations abolish the altered phase-separating behaviors

**Figure 4:**
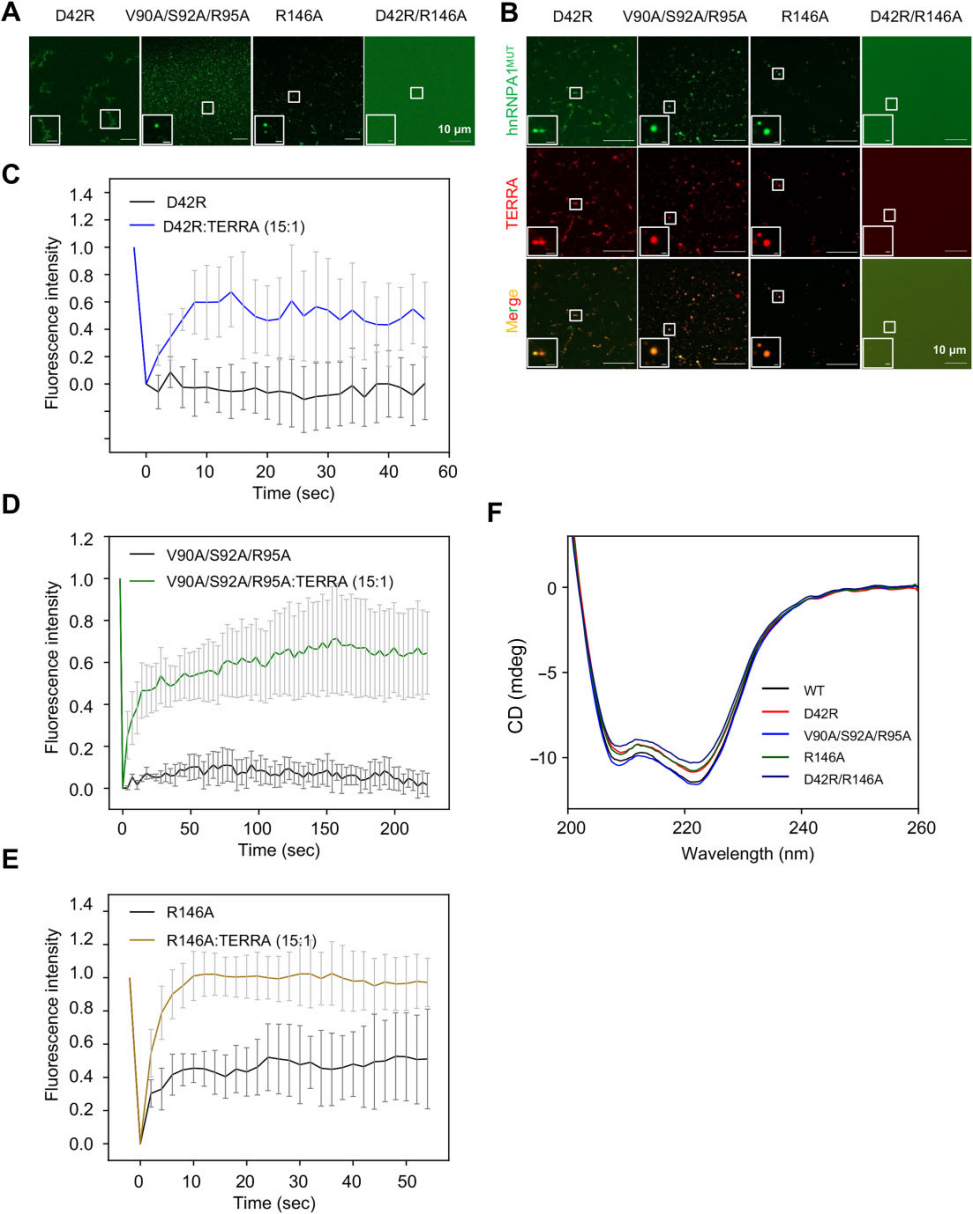
LLPS analysis of different hnRNPA1 variants *in vitro*. (**A** and **B**) Phase separation analysis of different hnRNPA1 variants in the absence (**A**) or presence (**B**) of a Cy3-labeled TERRA fragment using fluorescence microscopy. Protein was partially (5%) labeled with Alexa Fluor 488. The molar ratio of hnRNPA1 to TERRA was 30:1. Scale bar, 10 μm. (**C**–**E**) FRAP analysis of different hnRNPA1 variants with or without TERRA. (**F**) Circular dichroism (CD) results for wild-type and mutant hnRNPA1.

Next, we examined the phase-separating ability of hnRNPA1 variants with different key residue mutations in the presence or absence of TERRA (Figure 4A and B). As expected, the double mutant (D42R/R146A) that cannot interact with TERRA at all could not form granules either by itself or with TERRA, while the granule formation of the triple mutant (V90A/R92A/S95A), which mildly reduces the interaction between hnRNPA1 and RNA, was little affected. Unexpectedly, the phase-separating behavior of the mutants with single mutation within either of the two RRM domains was altered significantly even without RNA substrates, especially the D42R mutant, which produced aggregates. However, adding TERRA rescued fluidic granule formation much more significantly (Figure 4C–E), probably because the release of partial binding domains between hnRNPA1 and TERRA leads to the alternation of multivalent weak interactions. In addition, the circular dichroism spectroscopy of these mutants demonstrated that the secondary structures of hnRNPA1 did not change among all point mutations (Figure 4F).

### hnRNPA1 and TERRA form droplets in the nucleus in vivo

**Figure 5:**
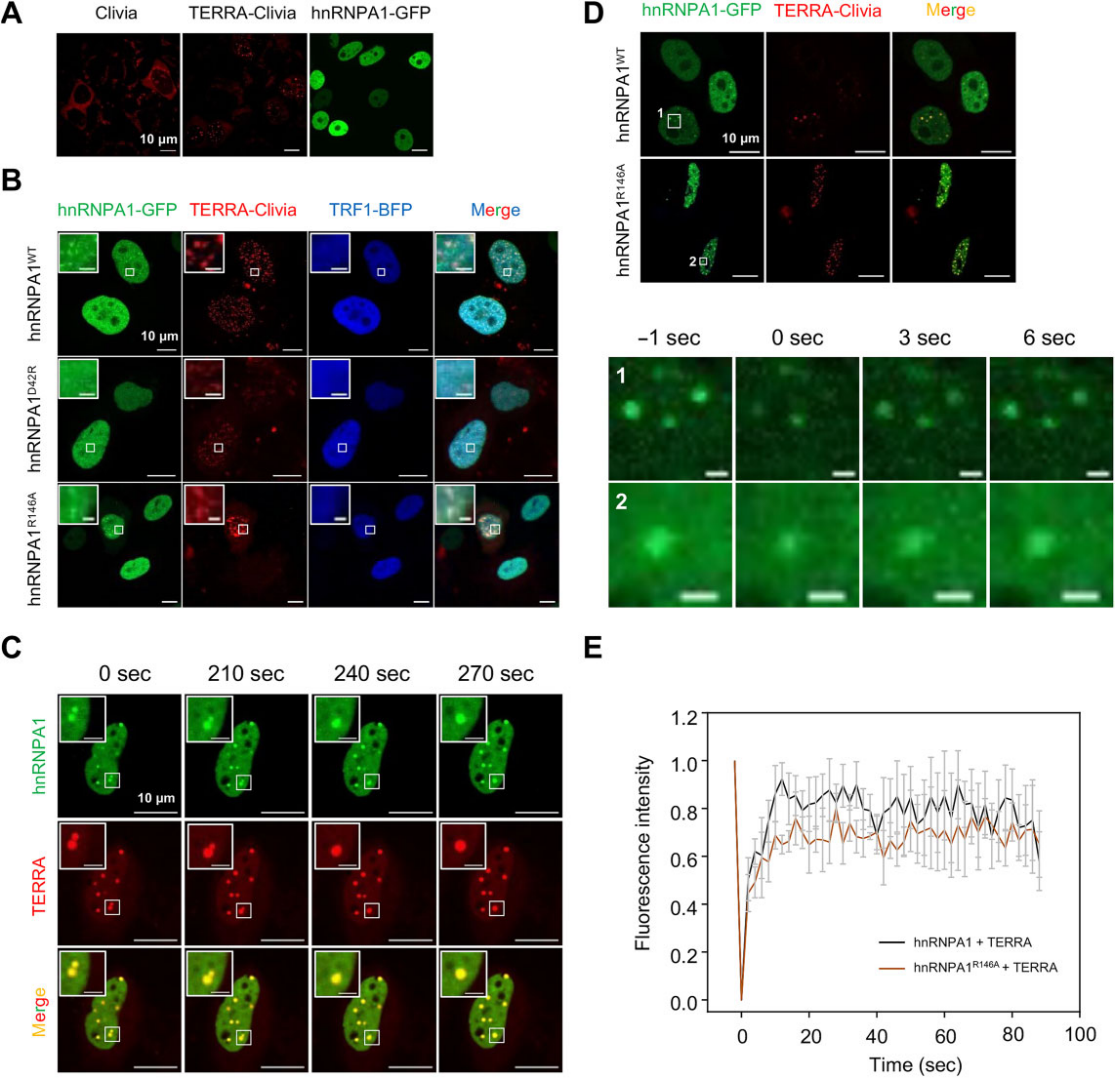
hnRNPA1 and TERRA form droplets in the nucleus in HeLa cells. (**A**) Live-cell imaging of overexpressed Clivia alone, TERRA-Clivia, or hnRNPA1-GFP in HeLa cells. Scale bar, 10 μm. (**B**) Colocalization of GFP-tagged wild-type or mutant hnRNPA1 with TERRA-Clivia and TRF1-BFP in the nucleus. Scale bar, 10 μm (original) or 1 μm (inset). (**C**) Phase-separated hnRNPA1–TERRA droplets fuse and form a larger droplet in the nucleus. Scale bar, 10 μm (original) or 1 μm (inset). (**D**) Snapshots of a bleached droplet of hnRNPA1^WT^ or hnRNPA1^R146A^ with TERRA in the nucleus. Scale bar, 10 μm (upper panel) or 1 μm (lower panel). (**E**) Quantification of average fluorescence intensities in **D**.

We further carried out *in vivo* experiments by overexpressing GFP-tagged hnRNPA1 protein and Clivia-tagged TERRA in HeLa and 293T cells, respectively. To investigate the behaviors of hnRNPA1 and TERRA near the telomere region, their expression needs to be tightly controlled to minimize the DNA damage response induced by overexpressing TERRA or the nuclear export of hnRNPA1 due to the stress response. Therefore, we first overexpressed hnRNPA1-GFP and TERRA-Clivia individually in HeLa cells to observe their subcellular distributions. The green fluorescence of hnRNPA1-GFP was evenly distributed in the nucleus (Figure 5A). Intriguingly, we observed numerous red puncta of TERRA-Clivia in the nucleus, which was not found in the cells expressing Clivia fluorogenic RNA alone (Figure 5A), indicating puncta aggregation of TERRA in the nucleus. When co-overexpressed, hnRNPA1 and TERRA could phase-separate and form liquid puncta, but these hnRNPA1–TERRA puncta did not colocalize with the telomere region, labeled by the TRF1 protein (Figure 5B), suggesting that the purpose of forming granules is to regulate the localization of hnRNPA1 and TERRA in the nucleus. Similar results were observed in 293T cells (Supplementary Figure S3), demonstrating that the regulation of localization via forming hnRNPA1–TERRA granules is a common mechanism.

To check the fluidity of the hnRNPA1–TERRA puncta in live cells, we carried out consecutive imaging of the puncta and observed the fusion process of two adjacent small condensates merging into a larger one (Figure 5C). We then overexpressed the identified hnRNPA1 variants in HeLa cells. hnRNPA1^R146A^ still formed droplets but with lower number and smaller size, whereas hnRNPA1^D42R^ completely lost the ability to form droplets (Figure 5B), consistent with that the binding affinity between hnRNPA1^D42R^ and TERRA is ∼1/3 of that between hnRNPA1^R146A^ and TERRA. FRAP assays also demonstrated that both hnRNPA1^WT^–TERRA and hnRNPA1^R146A^–TERRA condensates were highly dynamic (Figure 5D and E). However, the fluorescence recovery of hnRNPA1^R146A^–TERRA granules was slower, probably due to the interrupted interaction between RRM2 and the corresponding TERRA copy.

Furthermore, the co-phase separation of hnRNPA1 and TERRA was examined in a typical ALT-associated PML body cell line U2OS (Supplementary Figure S4). Similarly, hnRNPA1 and TERRA formed granules in the nucleus, which were fluidic and exhibited fast recovery after photobleaching but could not colocalize with TRF1-labeled telomere region. Expression of D42R mutant completely disrupted the hnRNPA1–TERRA granule formation in U2OS cells (Supplementary Figure S4A). Thus, we conclude that hnRNPA1 recognizes TERRA to regulate genomic stability and telomere capping both *in vitro* and *in vivo.*

## Discussion

The most critical procedure of telomeric stability maintenance is to regulate and guide orderly telomere replication and proper capping. One eukaryotic ssDNA-binding protein, RPA, attracted our interest because it is responsible for both DNA replication and DNA repair processes (Wold, 1997; Sui et al., 2022). The disassociation of RPA from each telomeric overhang at the telomere is critical for chromatin duplication in S phase, as the accumulation of RPA-coated ssDNA is harmful and triggers the ATR checkpoint pathway to promote DNA repair (Zou and Elledge, 2003; Zou, 2007). Thus, hnRNPA1 replaces RPA by competitively binding with telomeric ssDNA. Furthermore, hnRNPA1 is antagonized by TERRA, which is a long noncoding RNA that is transcribed from telomeres and subsequently promotes the recruitment of POT1 to complete the shelterin complex assembly and telomere organization (Flynn et al., 2011). These sequential procedures are well-regulated and coordinate with the mitotic cycles. As previously reported, the TERRA amount descends dramatically from the late S phase to the early G2 phase and increases again when cells have passed through G2 and M phases and reenter the subsequent G1 phase, reaching the initial expression level (Porro et al., 2010). Therefore, how hnRNPA1 and TERRA leave telomeres and guide the loading of POT1 synchronously and rapidly at all 92 chromatin ends in humans is still unclear. A recent study reported that PP2A-dependent hnRNPA1 dephosphorylation and TERRA accumulation facilitate the protective capping structure of newly replicated telomeres (Sui et al., 2022). In our study, by revealing the molecular details of the hnRNPA1–TERRA interaction and their co-phase separation *in vitro* and *in vivo*, we provided new insight into how the phase separation of hnRNPA1 and TERRA contributes to the process of hnRNPA1 leaving from the telomere and thus promotes the telomeric capping.

We also noticed that the condensates formed by hnRNPA1 and TERRA were much more fluidic than those by hnRNPA1 alone. According to the crystal structure we solved, hnRNPA1 forms a 2:2 complex with TERRA through the UP1 domain, which could increase the valency between hnRNPA1 and TERRA and thus lead to more gel-like and less fluidic granules. However, the experimental results indicated the opposite: TERRA lowers the viscosity and accelerates the fluidity of hnRNPA1 granules, similar to the phase separation of the LAF-1 protein (Elbaum-Garfinkle et al., 2015). On the other hand, the granule formed by arginine-rich dipeptide repeat proteins, which is the primary cause of amyotrophic lateral sclerosis, shows reduced fluidity while its multivalency increases (White et al., 2019). Therefore, the relationship between multivalency and liquid fluidity is still poorly understood. Nevertheless, the increased valency weakens the boundary of the granule to the dilute phase, which results in the dissociation of hnRNPA1 and TERRA from the telomere region quickly and synergistically in the S-to-G2 stage and ensures genome stability during the life cycle. It would be of great interest to examine the stoichiometry of hnRNPA1 and TERRA network during cell division cycle in real-time using the recently established super-resolution and tetrameric FRET imaging analyses (Xia et al., 2014; Song et al., 2023; Dou et al., 2024; Yang et al., 2024).

In summary, our findings reveal the molecular structure through which the hnRNPA1 UP1 domain interacts with TERRA. We reason that the phase separation-driven interaction between hnRNPA1 and TERRA likely guides genomic stability during the cell cycle.

## Materials and methods

### Cloning

For protein expression, DNA fragments encoding human full-length hnRNPA1 (residues 1–320) and UP1 (residues 1–196) were amplified by PCR using human brain and spinal cord tissues. The full-length hnRNPA1 and mutants were inserted into the pET-28a vector to produce the maltose-binding protein (MBP) tag-fused recombinant proteins with a PreScission cleavage site. UP1 and its mutants were cloned and inserted into the modified pET-22b vector with a His6 tag at the C-terminus.

For live-cell imaging, the GFP or mCherry gene was fused to the 3′ end of target genes to express GFP- or Cherry-fused proteins. Full-length hnRNPA1 (residues 1–320) and mutants were cloned and inserted into the pcDNA3.1 vector to produce GFP tag-fused recombinant proteins. TRF1 was inserted into the pcDNA3.1 vector, engineered to contain an N-terminal BFP tag. The cDNAs encoding tRNA-Clivia were inserted into the original sgRNA expression plasmid using the pEASY-Basic Seamless Cloning and Assembly Kit, which was linearized by PCR amplification to remove the native sgRNA scaffold. For imaging of diverse noncoding RNA TERRA, cDNAs of the 5′-UUAGGGUUAGGG-3′ fragment were synthesized and inserted into tRNA-Clivia. The linearized fragments were phosphatized and ligated to generate plasmids expressing chimeric (UUAGGG)_2_ TERRA snRNA.

### Protein expression and purification

Full-length hnRNPA1 (wild-type or mutant) was expressed with an N-terminal His6-MBP tag in *Escherichia coli* strain BL21(DE3) cells in LB medium containing 50 μg/ml kanamycin at 16°C overnight, and protein expression was induced by 0.20 mM (final concentration) isopropyl-β-D-thiogalactopyranoside (IPTG) at OD_600_ 0.5–0.6. Recombinant proteins were purified using a Ni^2+^-chelating column (GE Healthcare) followed by size-exclusion chromatography (Superdex 200 column, GE Healthcare) with column buffer containing 20 mM HEPES (pH 7.5) and 1 M NaCl. The purified hnRNPA1 protein was diluted in buffer A (20 mM HEPES, pH 7.5, and 150 mM NaCl).

For UP1 and mutants, cells were grown to OD_600_ 0.8 at 37°C in LB medium with 100 mg/ml ampicillin. *E. coli* cells expressing proteins were induced with 0.5 mM IPTG at 37°C for 5 h, spun down at 5000 rpm, resuspended in lysis buffer (20 mM Tris–HCl, pH 7.5, and 1 M NaCl), and lysed with a high-pressure homogenizer before centrifugation. The supernatants were first purified using a His Trap column (GE Healthcare), followed by size-exclusion chromatography on a Hiload 16/60 Superdex 75 column (GE Healthcare). All purified proteins were changed to a buffer containing 20 mM Tris–HCl (pH 7.5), 150 mM NaCl, and 0.2 mM EDTA for the following experiments.

### Crystallization, data collection, and structure determination

The UP1 protein was concentrated to 10 mg/ml and mixed with 5′-UUAGGGUUAGGG-3′ at a 1:1.2 molar ratio for binary complex crystallization. Crystals were grown for a month using the sitting drop vapor diffusion method at 291 K. Crystals were refined in the buffer containing 100 mM HEPES (pH 7.0), 10% (*w*/*v*) PEG4000, and 10% isopropanol.

All data were collected with the Shanghai Synchrotron Radiation Facility (SSRF) beamline BL18U1. Data intensity was indexed and scaled by HKL2000. The UP1–TERRA complex was solved by molecular replacement using the program Phaser in CCP4. The structure of the UP1–telomeric DNA complex (PDB code: 2UP1) was used as an initial model. Structural refinements were performed using REFMAC5 and PHENIX. Structure figures were generated by PyMOL. Experimental structure factors and the coordinates of the final model have been deposited into the Protein Data Bank (PDB) with the access code 8X0N. Crystal diffraction data and refinement statistics are displayed in Table 1.

### ITC experiment

ITC experiments were carried out using a MrcroCal PEAQ ITC instrument (Malvern Panalytical) at 20°C with a syringe protein concentration of 160 μM and a cell RNA concentration of 10 μM. Proteins and RNA samples were prepared by buffer exchange in 20 mM Tris–HCl (pH 7.5) and 150 mM NaCl. Thermodynamic analyses were performed by titrations of protein (syringe samples) into cell protein, with an initial injection of 1 μl followed by 19 consecutive injections of 2 μl, each separated by a time interval of 120 sec. The binding isotherms were integrated to give the enthalpy change ΔH plotted as a function of the protein and RNA molar ratio. The initial titration point was always discarded.

### Fluorescence labeling of protein

Alexa Fluor 488 NHS ester (Thermo Fisher) and Alexa Fluor 561 NHS ester (Thermo Fisher) were dissolved in dimethyl sulfoxide. Highly purified proteins were prepared in buffer A. The protein was first passed through a Hi-Trap desalting column (GE Healthcare, 17-0851-01) to ensure that any residual reducing agents were removed. Then, the protein sample was incubated with the dye overnight at 4°C (fluorophore-to-protein molar ratio 1:1). The fluorophores and other small molecules were removed from the proteins by passing the reaction mixture through the desalting column with buffer A.

### Confocal microscopy for in vitro droplet formation

In imaging assays, fluorescence-labeled proteins were diluted with the corresponding unlabeled proteins in the same buffer. All proteins were prepared in buffer A. The MBP tag of the fusion protein was removed with PreScission protease and the protein was diluted in buffer B (20 mM HEPES, pH 7.5) to a final hnRNPA1 concentration of 30 μM. DNA or RNA was dissolved in buffer B and mixed with protein before imaging. The samples were dropped on a glass slide.

For imaging, droplets were observed either on a glass slide or in a glass-bottom cell culture dish for differential interference contrast or fluorescence imaging (Imager M2 from Carl Zeiss and Zeiss LSM 980).

### FRAP assay

At room temperature, FRAP assay was performed with a confocal microscope (Zeiss LSM 980 Meta plus Zeiss Axiovert zoom) with a 63× oil objective and a 488/633 laser line. All proteins were prepared in buffer A. Unlabeled MBP-hnRNPA1 was mixed with fluorophore-labeled proteins at a molar ratio of 1:1. The MBP tag of the fusion protein was removed with PreScission protease and the protein was diluted in buffer B to a hnRNPA1 concentration of 30 μM. After blending, the samples (10 μl each) were loaded on an ultrathin glass-bottom dish. Defined regions were photobleached at specific wavelengths, and the fluorescence intensities in these regions were collected every 2 sec (for *in vitro* droplets) and normalized to the initial intensity before bleaching. Image intensity was measured by mean region of interest and further analyzed by Origin.

### Cell culture and plasmid transfections

293T and HeLa cells were cultured in Dulbecco's Modified Eagle Medium (DMEM) containing 10% fetal bovine serum and 1% penicillin/streptomycin (HyClone). Cells were grown at 37°C in a humidified incubator with 5% CO_2_ and passaged 1–2 times per week.

Cells were seeded in a 4-chamber glass-bottom dish for confocal imaging. When the cells reached 50% confluence, plasmids were transfected into cells using Hieff Trans® Universal Transfection Reagent (Yeasen, 40808ES02). The final concentration of plasmids was 1.0 mg/ml. Twenty-four hours after transfection, the cells were imaged using a confocal microscope.

### Live-cell imaging

Live-cell imaging was performed on an inverted Zeiss LSM 980 AxioObserver confocal microscope equipped with a motorized stage, a full incubation chamber maintaining 37°C/5% CO_2_, a heated stage, an X-Cite 120 illumination source, and several laser lines. Before imaging, the cells were treated with DMEM containing 0.5 μM NBSI618 (FR Biotechnology) for 10 min at 4°C to label the Clivia-tagged TERRA as reported before (Jiang et al., 2023). hnRNPA1 and TERRA were detected using 488-nm and 543-nm lasers, respectively. TRF1 was detected using a 405-nm laser. A single confocal plane was maintained, and images were collected for 30 sec for the fusion image. Defined regions were photobleached using the 488-nm laser, and the fluorescence intensities in these regions were collected every 3 sec and normalized to the initial intensity before bleaching. Images were processed using ImageJ (NIH).

## Supplementary Material

mjae037_Supplemental_File

## Notes

**Author contributions:** Z.X., Y.S., and L.Z. conceived and designed this study; Z.X. and L.Z. performed most of the experiments and analyzed the results; Y.L. and X.X. contributed to part of the experiments; F.L., Y.Y., H.Z., F.M., X.L., and X.C. provided advice and assistance for this study; Z.X., X.C., Y.S., and L.Z. wrote the manuscript.
