# Supplementary material for: Phase separation of hnRNPA1 and TERRA regulates telomeric stability
Source: J Mol Cell Biol. 2024 Sep 23;16(9):mjae037. doi: 10.1093/jmcb/mjae037 (PMC12019227; doi:10.1093/jmcb/mjae037)
Supplement: mjae037_Supplemental_File [file mjae037_supplemental_file.pdf]

Phase separation of hnRNPA1 and TERRA regulates telomeric stability

Ziyan Xu[1,2,3], Yongrui Liu[1,2,3], Fudong Li[1,2,3], Yi Yang[4], Hong Zhang[5,6], Feilong Meng[7], Xing Liu[1,2,3], Xin Xie[4,*], Xianjun Chen[4,*], Yunyu Shi[1,2,3,*], and Liang Zhang[1,2,3,*]

[1] Center for Advanced Interdisciplinary Science and Biomedicine of IHM, Division of Life Sciences and Medicine, University of Science and Technology of China, Hefei 230027, China

[2] Ministry of Education Key Laboratory for Membraneless Organelles and Cellular Dynamics, Division of Life Sciences and Medicine, University of Science and Technology of China, Hefei 230027, China

[3] Hefei National Research Center for Cross-disciplinary Science, Division of Life Sciences and Medicine, University of Science and Technology of China, Hefei 230027, China

[4] Optogenetics and Synthetic Biology Interdisciplinary Research Center, State Key Laboratory of Bioreactor Engineering, East China University of Science and Technology, Shanghai 200237, China

[5] National Laboratory of Biomacromolecules, CAS Center for Excellence in Biomacromolecules, Institute of Biophysics, Chinese Academy of Sciences, Beijing 100101, China

[6] College of Life Sciences, University of Chinese Academy of Sciences, Beijing 100049, China

[7] State Key Laboratory of Molecular Biology, Shanghai Institute of Biochemistry and Cell Biology, Center for Excellence in Molecular Cell Science, Chinese Academy of Sciences, University of Chinese Academy of Sciences, Shanghai 200031, China

* Correspondence to: Liang Zhang, E-mail: zhangl99@ustc.edu.cn ; Yunyu Shi, E-mail: yyshi@ustc.edu.cn ; Xianjun Chen, E-mail: xianjunchen@ecust.edu.cn; Xin Xie, E-mail: Y30150272@mail.ecust.edu.cn

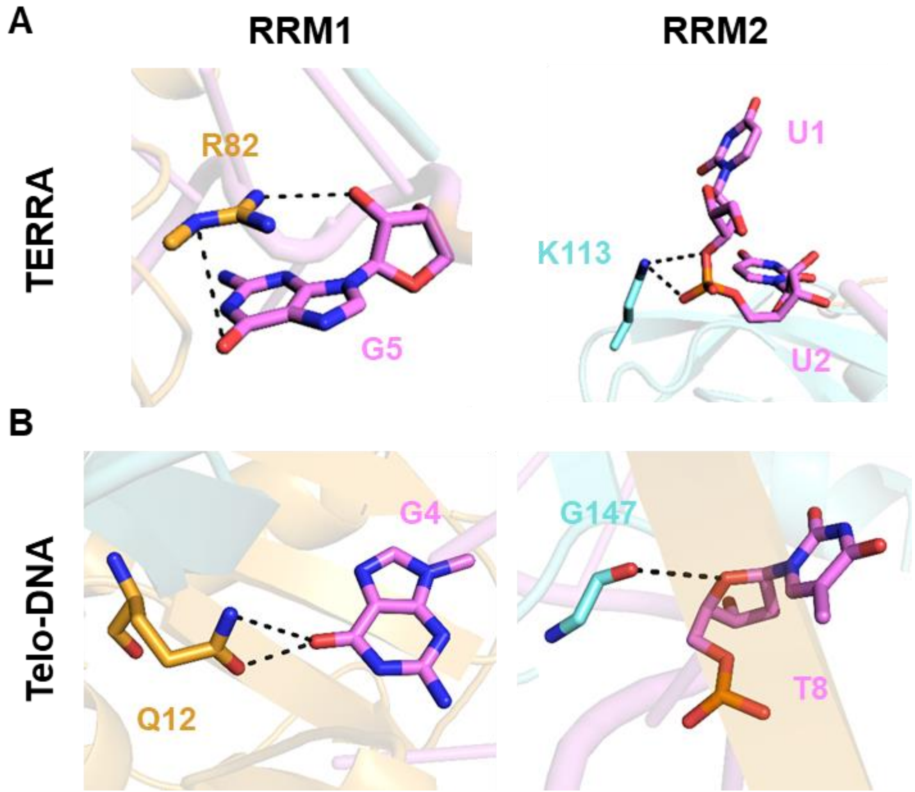

Supplementary Figure S1. Structural basis of UP1 recognitions with the TERRA RNA fragment and Telomeric DNA (Telo-DNA) fragment. A. The interaction details of TERRA with Arg82 (left) and Lys113 (right) of UP1 in the complex structure of UP1-TERRA. **B.** The interaction details of Telo-DNA with Gln12 (left) and Gly147 (right) of UP1 in the complex structure of UP1-TeloDNA.

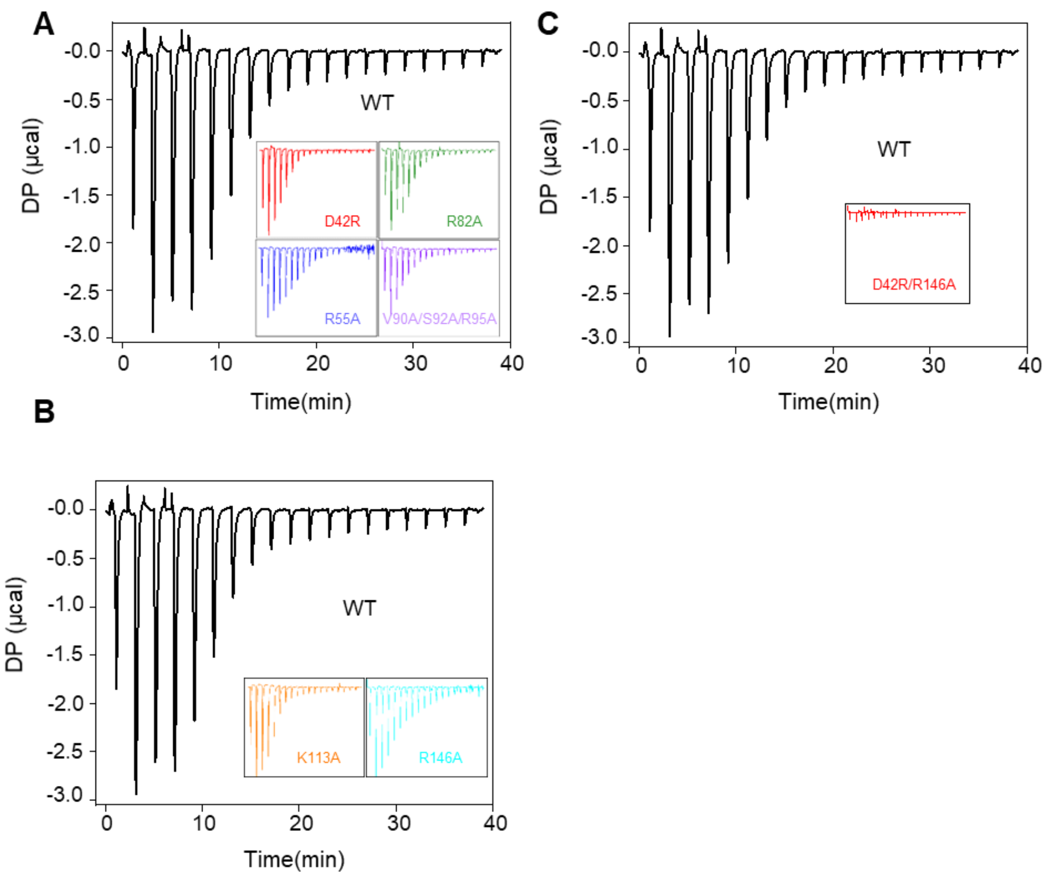

Supplementary Figure S2. ITC results for UP1 wild-type or mutations to a 12 bp TERRA. (A). The wild-type (black) or mutations (D42R as red, R55A as blue, R82A as green or V90A/S92A/R95A triple mutations as purple) of RRM1 from UP1. (B) Wild-type (black) or mutant (K113A as orange or R146A as cyan) RRM2 from UP1. (C). The wild-type (black) or double mutations (D42R/R146A as red) of both RRMs from UP1.

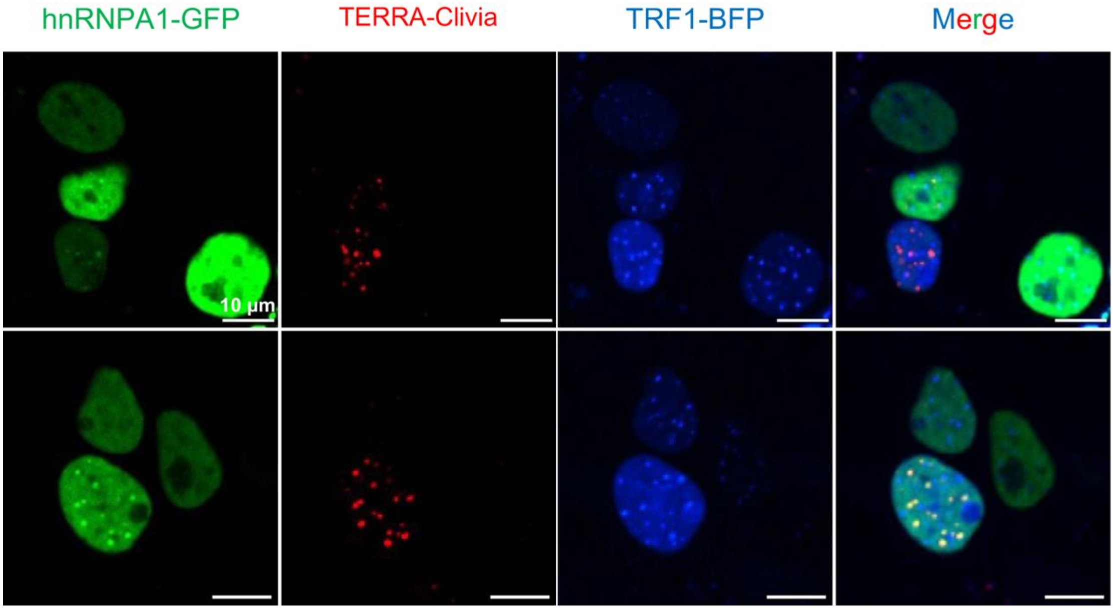

Supplementary Figure S3. Droplets of hnRNPA1 and TERRA formed in the nucleus in 293T cells.

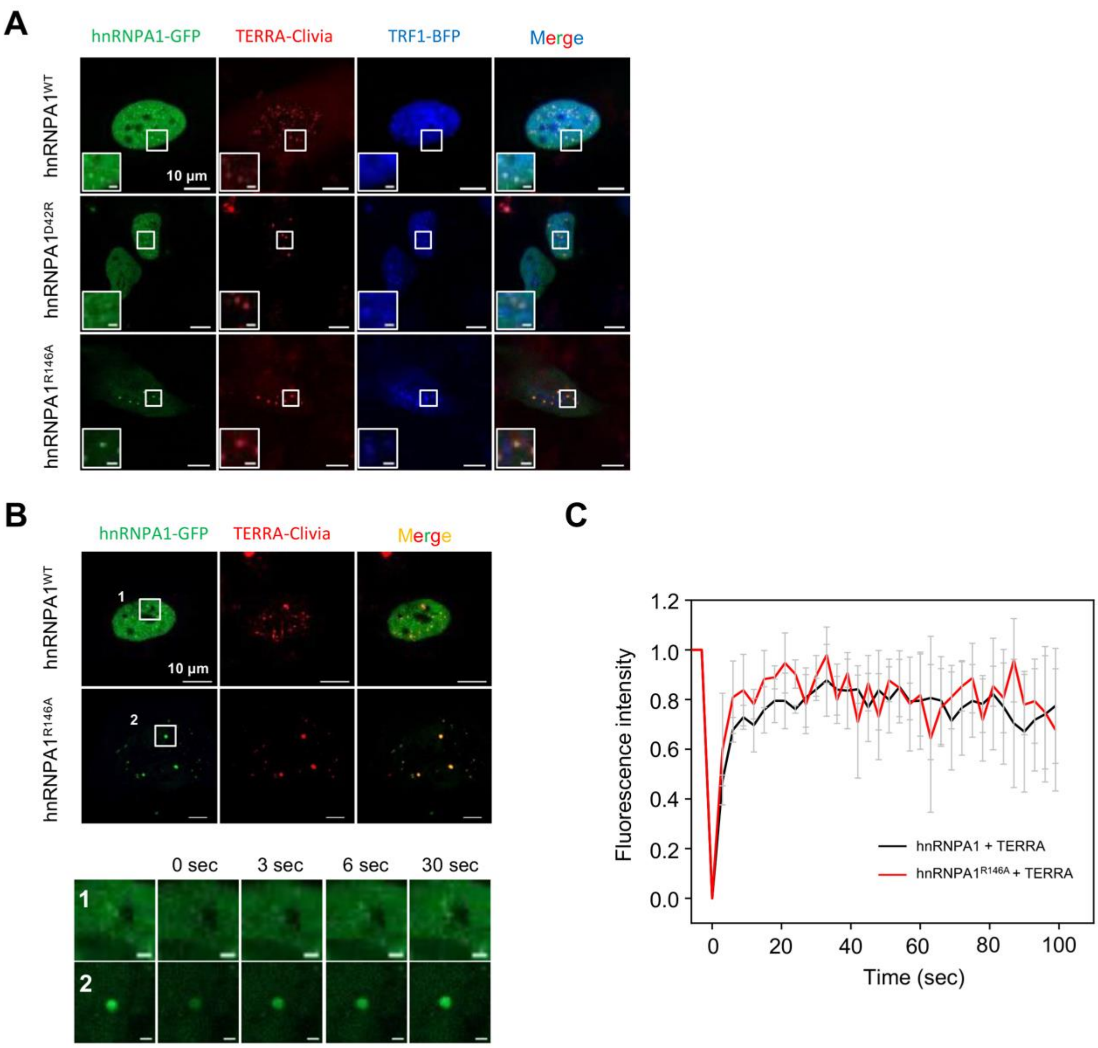

Supplementary Figure S4. HnRNPA1 and TERRA form droplets in the nucleus of U2OS cells. A. Observation of colocalization of GFP-tagged hnRNPA1 wild-type or mutants with TERRA-Clivia and TRF1-BFP in the nucleus. **B.** Snapshots of a bleached droplet of hnRNPA1 or hnRNPA1^{R146A} with TERRA in the nucleus. **C.** Quantification of average fluorescence intensities of panel B. Scale bars of panels A and B are 10 μm except the enlarged images, the scale bar for the left corner enlarged images in panel A and beneath the panel B are 1 μm.
